# Editorial: Community series in mycoviruses and related viruses infecting fungi, lower eukaryotes, plants and insects, volume II

**DOI:** 10.3389/fmicb.2024.1349526

**Published:** 2024-01-31

**Authors:** Hiromitsu Moriyama, Ioly Kotta-Loizou, Kook-Hyung Kim

**Affiliations:** ^1^Laboratory of Molecular and Cellular Biology, Graduate School of Agriculture, Tokyo University of Agriculture and Technology, Fuchu, Japan; ^2^Department of Clinical, Pharmaceutical and Biological Science, School of Life and Medical Sciences, University of Hertfordshire, Hatfield, United Kingdom; ^3^Department of Life Sciences, Faculty of Natural Sciences, Imperial College London, London, United Kingdom; ^4^Department of Agricultural Biotechnology, College of Agriculture and Life Sciences, Plant Genomics and Breeding Institute, Research Institute of Agriculture and Life Sciences, Seoul National University, Seoul, Republic of Korea

**Keywords:** mycovirus, *Phytophthora*, *Metarhizium*, *Talaromyces*, *Aspergillus*, RNA silencing, antiviral drugs

The Frontiers Community Series in “*Mycoviruses and related viruses infecting fungi, lower eukaryotes, plants and insects, volume II*” was initiated following the success of the Frontiers Research Topic “*Mycoviruses and related viruses infecting fungi, lower eukaryotes, plants and insects*.” Unlike the original Research Topic, which accepted submissions through both *Frontiers in Microbiology* and *Frontiers in Plant Pathology*, the Community Series was limited to *Frontiers in Microbiology* and covered exclusively one section: *Virology*. As a result, most manuscripts in the Community Series focused on viruses infecting insects and human pathogenic fungi, instead of plant pathogens. Our Community Series accommodates 5 high-quality Original Research manuscripts that provide insight into the biology of a range of viruses found in oomycetes and filamentous fungi, including the discovery and characterization of two novel members of the family *Polymycoviridae*, mycovirus elimination, and numerous host-virus interactions and virus-mediated phenotypes.

Sakuta et al. investigated at the molecular level two members of the family *Endornaviridae*, Phytophthora endornavirus 2 and 3, found in the plant pathogenic oomycetes *Phytophthora* rot of asparagus. These endornaviruses were shown to be present in the cells primarily as positive-sense, singe-stranded RNA and have 5′ terminal nick structures approximately 1 kb in length. Full-length cDNA clones of the endornaviruses were introduced into the model yeast *Saccharomyces cerevisiae* under the control of both constitutive and inducible promoters, and their overexpression resulted in reduced yeast growth. Tagging endornavirus proteins with green fluorescent protein allowed their visualization within yeast cells.

Wang et al. reported the sequence of a new member of the family *Polymycoviridae*, Metarhizium anisopliae polymycovirus 1, from the insect-pathogenic ascomycete and popular biocontrol agent *Metarhizium anisopliae*. Polymycovirus infection increases fungal growth, conidiation, and sensitivity to ultraviolet irradiation by altering host gene expression but has no significant effect on virulence. The polymycovirus-mediated phenotypes were not linked to individual polymycoviral proteins.

Teng et al. reported the sequence of a new member of the family *Polymycoviridae*, Talaromyces amestolkiae polymycovirus 1, the first mycovirus found to infect *Talaromyces amestolkiae*, an ascomycete ubiquitous in air, soil, food, and plants that is also an opportunistic human pathogen. Polymycovirus infection reduced the production of the characteristic red pigments of *T. amestolkiae*, while inducing clustering of fungal sclerotia. Transcriptome profiling illustrated that polymycovirus infection results in the downregulation of host genes associated with metabolism.

Jiang et al. investigated the role of RNA interference as an antiviral defense against Aspergillus flavus partitivirus 1 in *Aspergillus flavus*, an ascomycete known for aflatoxin production. Partitivirus infection depleted sclerotia production and induced expression of genes encoding fungal enzymes, including Dicer, Argonaut, and RNA-dependent RNA polymerases, resulting in virus-derived small RNAs. Deletions of these genes affected viral RNA levels, fungal sporulation, and host sensitivity to cell wall, genotoxic, osmotic, and oxidative stress.

Ikeda et al. explored alternatives for elimination of RNA viruses using antiviral drugs, a process generating virus-infected and virus-free isogenic lines required for further phenotypic comparisons. The nucleoside analog 2′-C-methylcytidine was shown to be active against a wider range of mycoviruses as compared to ribavirin, 2′-C-methyladenosine and 7-Deaza-2′-C-methyladenosine and eliminated members of the families *Chrysoviridae, Mitoviridae, Partitiviridae*, and *Polymycoviridae* infecting the human pathogenic ascomycete *Aspergillus fumigatus*.

As Associate Editors, we would like to take this opportunity to thank and acknowledge all the contributing authors who chose our Community Series in “*Mycoviruses and related viruses infecting fungi, lower eukaryotes, plants and insects, volume II*” as a vehicle for sharing their exciting work.

## Author contributions

HM: Writing – review & editing. IK-L: Writing – original draft. K-HK: Writing – review & editing.

